# Resolutions of Respect to Dr. Eames

**Published:** 1894-06

**Authors:** 


### Obituary.

### RESOLUTIONS OF RESPECT TO DR. EAMES.

THE St. Louis Dental Society met on May 1 at the Missouri Dental College. During the meeting the following resolutions were adopted:

*Resolved*, That in the death of Dr. W. H. Eames this Society has lost a valuable member, one deeply interested in the progress of his profession, and for years a prominent supporter and contributor to its leading journals. This Society gratefully acknowledges his services in advancing its interests and preserving the high standard of dentistry. We extend our heartfelt sympathies to his bereaved family, and mourn with them the loss of a devoted husband and father, our brother, of whom it can be truly said, that those who knew him best loved him best.

*Resolved*, That copies of these resolutions be furnished to the dental journals for publication and a copy be sent to the bereaved family.

A. H. FULLER.
JOHN. G. HARPER.
WM. N. MORRISON.

